# Effective Energy Transfer via Plasmon-Activated High-Energy Water Promotes Its Fundamental Activities of Solubility, Ionic Conductivity, and Extraction at Room Temperature

**DOI:** 10.1038/srep18152

**Published:** 2015-12-10

**Authors:** Chih-Ping Yang, Hsiao-Chien Chen, Ching-Chiung Wang, Po-Wei Tsai, Chia-Wen Ho, Yu-Chuan Liu

**Affiliations:** 1 Graduate Institute of Medical Science, College of Medicine, Taipei Medical University, 250 Wuxing St., Taipei 11031, Taiwan; 2 Department of Biochemistry and Molecular Cell Biology, School of Medicine, College of Medicine, Taipei Medical University, 250 Wuxing St., Taipei 11031, Taiwan; 3 School of Pharmacy, College of Pharmacy, Taipei Medical University, 250 Wuxing St., Taipei 11031, Taiwan; 4 Center for Cancer Research, Taipei Medical University, 250 Wuxing St., Taipei 11031, Taiwan; 5 Biomedical Mass Imaging Research Center, Taipei Medical University, 250 Wuxing St., Taipei 11031, Taiwan

## Abstract

Water is a ubiquitous solvent in biological, physical, and chemical processes. Unique properties of water result from water’s tetrahedral hydrogen-bonded (HB) network (THBN). The original THBN is destroyed when water is confined in a nanosized environment or localized at interfaces, resulting in corresponding changes in HB-dependent properties. In this work, we present an innovative idea to validate the reserve energy of high-energy water and applications of high-energy water to promote water’s fundamental activities of solubility, ionic conductivity, and extraction at room temperature. High-energy water with reduced HBs was created by utilizing hot electrons with energies from the decay of surface plasmon excited at gold (Au) nanoparticles (NPs). Compared to conventional deionized (DI) water, solubilities of alkali metal-chloride salts in high-energy water were significantly increased, especially for salts that release heat when dissolved. The ionic conductivity of NaCl in high-energy water was also markedly higher, especially when the electrolyte’s concentration was extremely low. In addition, antioxidative components, such as polyphenols and 2,3,5,4’-tetrahydroxystilbene-2-O-beta-d-glucoside (THSG) from teas, and *Polygonum multiflorum* (PM), could more effectively be extracted using high-energy water. These results demonstrate that high-energy water has emerged as a promising innovative solvent for promoting water’s fundamental activities via effective energy transfer.

Liquid water with defects deviates from the perfect tetrahedral symmetry of a hydrogen-bond (HB) network, thereby allowing it to play a central role in a vast number of physical and chemical processes. Intramolecular HBs of water clusters and HBs with other molecules critically influence water’s fundamental reactivities^1^,^2^,^3^. Recently, unique properties of water with a distinguishable HB structure at interfaces have garnered wide attention^4^,^5^,^6^. However, these interesting properties of water are only found within the interfacial phase, limiting their applications. As reported by Velasco-Velez *et al.*, analysis of an ab initio molecular dynamic (AIMD) simulation revealed that ~50% of interfacial water molecules lie flat on the gold (Au) surface with broken HBs. This population of broken HB molecules is substantially higher than the 22% found in bulk water^7^. Water is a ubiquitous solvent in life sciences and physical and chemical reactions. In red corpuscles, aquaporin water channels are so selective that water passes through them to transfer nutrients and metabolites, while protons do not pass through^8^. In aqueous solutions, water can catalyze proton-transfer tautomerism^9^. In the solution process, the solubility of solid solutes in water is a typical issue of concern. In most cases, the solubility increases with increasing temperature; while the saturated solubility of NaCl is difficult to increase by elevating the temperature of the solution.

On the other hand, if the energy of a photon impinging on a noble metal surface is resonant with the frequency of the collective oscillations of surface plasmons, light absorption is dramatically increased. Nanostructured metal surface plasmons can be locally concentrated, from which the large absorption of energy generates excited electrons known as hot electrons^10^,^11^,^12^. Hot-electron transfer (HET) can promote many chemical reactions, including the dissociation of hydrogen^13^,^14^ and water splitting^15^,^16^, and can be developed as plasmoelectric devices^17^. Also, hot electrons can move into the MoS_2_ monolayer to induce a structural phase transition^18^, and the generated photocurrent generated from hot-electron injection of Au nanorods into ZnO nanowires can be detected by near-infrared (IR) photodetectors^19^. However, efficiently extracting hot electrons is very tough, with a reported efficiency of <1%^10^. In our previous report^20^, HET was innovatively utilized to create Au nanoparticle (NP)-treated (AuNT) water with reduced HBs. The values of degree of non-hydrogen-bonded water (DNHBW) for deionized (DI), AuNT and super small water cluster (SWC) waters are 21.29, 26.23 and 30.31%, respectively^20^. The super SWC water was obtained by irradiating DI water-wetted Au NPs supported on ceramic particles with laser light at 532 nm *in situ* Raman spectrum measurement. The created bulk liquid water is energetic and in an electron-doping state, shows promise in its innovative availability to increase the efficiency and safety of hemodialysis^21^. In this work, we present innovative applications of room-temperature high-energy bulk liquid water by promoting water’s fundamental activities of solubility, ionic conductivity, and extraction, which are never considered when using conventional DI water. Interestingly, different evaporation rates of DI water and AuNT water, and their salt-containing solutions are also discussed.

Many biological, physical, and chemical processes involve energy transfer^22^. Biological photosynthesis utilizes the energy of several light photons and can reserve this created energy for use in challenging chemical reactions^23^, but only one excited photon is available in general photocatalytic chemistry^24^. Recently, Ghosh *et al.*^25^ reported that subsequent excitation of radical anions accumulates sufficient energy to reduce stable aryl chlorides into aryl radicals. In addition, Hammarström^26^ reported accumulative charge separation for solar fuel production, in which coupling light-induced single electron transfers to multi-electron catalysis was presented. In our group^20^, HET from plasmon excitation of resonantly illuminated Au NPs under green light-emitting diodes (LEDs, with wavelength maxima centered at 530 nm) was innovatively utilized to create liquid AuNT water with reduced HBs. The created bulk AuNT water was pH-neutral and free-standing, and its temperature was close to room temperature after creation. The resulting AuNT water can scavenge free radicals and assist the reduction of Au salts, suggesting its electron-doping state. In addition, compared to DI water, the chemical potential of AuNT water was significantly higher, as revealed from an equation of the vapor pressure-dependent chemical potential^27^ based on its significantly higher vapor pressure at room temperature^20^. This suggests that energetic AuNT water with reduced HBs possesses higher activity, which would be available for promoting biological, physical, and chemical water-related processes. Moreover, the distinct properties of AuNT water compared to DI water can persist for at least 3 days^20^, suggesting its similarity to biological photosynthesis which can use reserved energies for challenging chemical reactions. In this work, the electron-doping state of AuNT water was further directly confirmed by observing obviously slanting droplets of AuNT water compared to DI water, which were sprayed from a stainless steel needle electrode (cathode) in an electron-spin module at 10 kV, toward a grounded counter electrode (anode) (Fig. S1). In stationary electric fields, most of the droplets of AuNT water from the cathodic needle were collected above the central axis of the anodic plate; while most of the droplets of DI water from the cathodic needle were collected below the central axis of the anodic plate. Moreover, more droplets of AuNT water, compared to DI water, were observed on the anodic plate. These phenomena suggest that electrostatic interactions between the negatively charged AuNT water and anodic plate were stronger.

First, we examined increased saturated solubilities of alkali metal-chloride salts in high-energy AuNT water with weak HBs, compared to conventional DI water. As shown in Fig. 1a, solubilities of alkali metal-chloride salts in water significantly increased when DI water (~pH 6.95) was replaced with AuNT water (~pH 6.96) at room temperature (~22.9 °C), especially for LiCl in an exothermic solution process. These findings are very interesting and important. The general strategy for increasing the solubility of a salt in water is to elevate the solution temperature. In this work, the innovative strategy of increasing the solubility of the salt utilizing energetic AuNT water without changing the temperature of the solution was encouraging. The solubility of LiCl (with a heat of solution of −38 kJ mol^−1^) significantly increased from 79.0 ± 1.97 to 106.5 ± 7.91 g dL^−1^ (an increase of ~35%) in AuNT water compared to DI water. The solubility of NaCl (with a heat of solution of 4 kJ mol^−1^) significantly increased from 35.7 ± 0.02 to 42.8 ± 0.52 g dL^−1^ (an increase of ~20%) in AuNT water compared to DI water. It is well known that it is difficult to increase the solubility of NaCl by elevating the temperature of the solution^28^,^29^ because its heat of solution is close to zero (an increase of ~1% from room temperature to 100 °C). Compared to DI water, a significant increase of ~20% solubility of NaCl in AuNT water would impact many biological processes because saline solutions are ubiquitously employed in medicine. Notably, the increase of solubility of KCl in a more obviously endothermic solution in AuNT water was slight. The solubility of KCl (with a heat of solution of 17 kJ mol^−1^) slightly increased from 33.0 ± 0.92 to 33.6 ± 0.72 g dL^−1^ (an increase of ~1.8%) in AuNT water compared to DI water. The dissolution of salt is a well-known physical process described in general textbooks based on DI water, which involves two kinds of lattice and hydration energies. The hydration of ions favors the dissolution of an ionic solid in water; while lattice energy holds ions together in a crystal lattice, and works against the solution process. Lattice energy minus hydration energy determines the heat of the solution. Generally, the solubility of a salt in exothermic solution processes is higher than that in endothermic solution processes. The corresponding energies of LiCl, NaCl, and KCl in solutions based on DI water are exhibited in the inserted table of Fig. 1a. The significant increase in the saturated solubility of LiCl in high-energy AuNT water is consistent with Le Chatelier’s principle. In the dissolution-crystallization equilibrium reaction, high-energy AuNT water can provide additional energy to favor the dissolution direction when dissolving releases considerable heat, because the released heat is readily removed to the ambient laboratory air. In solution, the attraction of water molecules to ions comes from the ion-dipole force. The O end of H_2_O orients toward the cation, whereas H atoms of H_2_O orient toward the anion. In conventional DI water with strong HBs, compared to water with a disordered HB structure at interfaces, the ion-dipole force is reduced due to a reduction in water’s polarity. In our system, the distinct nature of electron-doping AuNT water with a higher chemical potential and weaker HBs is responsible for its significant enhancement in solution activity with alkali metal-chloride salts. AuNT water with a higher chemical potential can provide supplementary energy to overcome the lattice energy in the solution process, more effectively pulling ions apart and thus favoring dissolution. Cavity high-energy water at the hydrophobic surface, where it cannot form a stable HB network, was also revealed from theoretical considerations^30^. In addition, the influence of water’s HBs on its reactivity was discussed in the HCl dissociation in small water, which involves energy transfer and the breaking of HBs^2^. AuNT water possessing weak HBs suggests that there are more free water molecules available for attraction of water molecules to ions, compared to DI water with strong HBs. This effect can increase the hydration energy, and similarly, more effectively pull ions apart, thus increasing the solubility. Consistently, the HB network geometry can influence reaction rates of molecules that are “solvated” in a cluster geometry of water, as reported in a theoretic approach^31^. As reported in probing water micro-solvation in proteins by water catalyzed proton-transfer tautomerism^9^ and in photobiocatalytic chemistry of oxidoreductases using water as the electron donor^32^, non-hydrogen-bonded O atoms of H_2_O with lone pairs of electrons contribute to these catalytic reactions. These interesting results that imply that free water molecules promote water’s activities are consistent with those proposed in this work.

Recently, halogen bonding (XB, the attractive interaction between an electron-deficient halogen atom and a Lewis base) in water, resulting in enhanced anion recognition in acyclic and rotaxane hosts, was reported in the literature^33^. This observation demonstrates the superior ability of XB over HB for strong anion binding in water. Since AuNT water used in this work is electron-doping, it can act as a Lewis base to provide additional attraction to Cl atoms of alkali metal-chloride salts. Therefore, increased solubility of chloride-containing salts was observed in AuNT water, compared to charge-neutral DI water. Also, the extreme surface propensity of halide ions in water was reported in the literature^34^. Concentrations of chloride and iodide at the water surface are higher than those in bulk water. On the other hand, the formation of non-HB water at the air/water interface was indeed probed using Raman multivariate curve resolution^35^. Integrating these findings, we concluded that non-HB water has an advantage over HB bulk water in dissolving salts, which is consistent with our findings that AuNT water with reduced HBs can dissolve more alkali metal-chloride salts. Generally, the saturated solubility of salt in water is inversely proportional to the heat of solution. The saturated solubility for exothermic solution processes is higher than that for endothermic solution processes due to entropic preferences. In this work, the lattice energy of salt was constant and independent of the kind of water used in the solution process. However, the hydration energy of salts in AuNT water significantly increased, as discussed before, resulting in a significant reduction in the sum (lattice energy minus hydration energy) of the heat of the solution. Therefore, a significant increase in the saturated solubility was observed in AuNT water instead of conventional DI water. In addition, both lattice and hydration energies are inversely proportional to the distance between neighboring ions, and this distance depends on the sum of the radii of the ions. For these reasons, these two kinds of energies decrease from the smallest LiCl to the largest KCl, as shown in the table inserted in Fig. 1a. Correspondingly, the effect of the increased hydration energy on the solubility of large KCl in AuNT water was reduced. Moreover, distinct attractions to large KCl from electron-doping AuNT water with weak HBs were reduced. These resulted in a slightly increased solubility of KCl in AuNT water.

Figure 1b shows the increased solubility of LiCl in aqueous solutions containing different ratios of AuNT water. It demonstrates a satisfactory linear relationship (*R*^2^ = 0.983) between LiCl solubility and the correspondingly increased content of AuNT water in solution. This result suggests that the mixing of AuNT water with DI water is a physical process, which does not change the distinct activity of AuNT water in solution.

Figure 2a shows ionic conductivities of NaCl at extremely low concentrations in DI water and in AuNT water at room temperature. Encouragingly, for pure water without NaCl electrolytes, the ionic conductivity of AuNT water (0.490 ± 0.004 μS cm^−1^) was ca. 6-fold the magnitude of that of DI water (0.078 ± 0.001 μS cm^−1^) although their conductivities in pure waters were both extremely low. However, the ionic conductivity of 3.125 × 10^−4^ N NaCl in AuNT water (2.74 ± 0.01 μS cm^−1^) was almost the same as that in DI water (2.71 ± 0.01 μS cm^−1^). Moreover, differences in ionic conductivities of NaCl in different waters were slight at NaCl concentrations of >3.125 × 10^−4^ N, whereas the increased ionic conductivity of NaCl in AuNT water was reduced with an increase in the NaCl concentration below 3.125 × 10^−4^ N. The ionic conductivity in water is generally governed by two factors: the abilities of ion diffusion and ion transfer. Based on these criteria, the ionic conductivity of a specific electrolyte with a constant concentration at room temperature in conventional DI water would be a constant value. Increasing the ionic conductivity in high-energy and electron-doping AuNT water with weak HBs was never expected before. In our previous study^21^, it was reported that AuNT water’s weak HBs were responsible for the correspondingly novel performance in solute diffusion, which is a critical factor for hemodialysis efficiency. The calculated diffusion coefficient of 30 mM K_3_Fe(CN)_6_ (involving a one-electron reaction) increased from 2.76 × 10^−6^ to 4.62 × 10^−6^ cm s^−1^ (an increase of ~67%) when using an AuNT water-based saline solution instead of a conventional DI water-based saline solution. The magnitude of the diffusion coefficient of 1 mM hydroquinone (involving a two-electron reaction) in an AuNT water-based saline solution also increased by ca. 24% (from 1.78 × 10^−6^ to 2.20 × 10^−6^ cm s^−1^). In this work, the higher ion diffusion in AuNT water was responsible for the correspondingly higher ionic conductivity of electrolytes in AuNT water. Moreover, transfers of cations and anions in AuNT water with reduced HBs were easier because there were more free non-HB water molecules available, which can provide electron holes and pairs for corresponding ion transfers. Interestingly, the increased ionic conductivity in AuNT water was only observed for NaCl concentrations of <3.125 × 10^−4^ N. Above this concentration, ion conductivities in AuNT water and DI water were almost the same, and the distinct properties of AuNT water did not affect the corresponding ionic conductivity. We think that the ions themselves predominantly contributed to the conductivity of water at high ion concentrations, whereas the increased ionic conductivity from the contribution of AuNT water was slight, compared to the intrinsically high conductivities in these cases. Nevertheless, the electrochemical reaction with a roughened Au electrode in 0.1 N NaCl-containing AuNT water, compared to the DI water system, was significantly enhanced, as discussed below.

Figure 2b,c show typical triangular voltammetric curves for anodic dissolution and cathodic redeposition of Au onto an Au substrate (in a predominant (111) orientation) in 0.1 N NaCl solutions based on DI water and AuNT water, respectively. In these experiments, the same Au substrate was used in both the DI water and AuNT water systems. These oxidation-reduction cycle (ORC) procedures for obtaining surface-enhanced Raman scattering (SERS)-active Au substrates are generally used in the literature^36^,^37^. Basically, the anodic dissolution of a metal substrate is easier in AuNT water-based solutions than in DI water-based solutions, which reflects the enhanced current density. In electrochemical reactions, the recorded current density is critically dependent on the kinetics and diffusion controls. This result suggests that 0.1 N NaCl electrolytes used in ORCs can diffuse more efficiently in AuNT water-based solutions under an electric field, which contributes to the observed higher currents at constant applied potentials. In addition, with scanning in CV experiments, the dissolved Au ions in anodic scans would be redeposited onto the Au substrate in cathodic scans. Therefore, the Au substrate is roughened with scanning. This results in increased surface area. Thus, the increased anodic peak currents with scanning in CV experiments were observed in both DI water-based and AuNT water-based solutions. Moreover, when comparing Fig. 2b,c for experiments performed in DI water and AuNT water, respectively, with the same Au substrate, it was found that the anodic dissolution of Au from the Au electrode in 0.1 N NaCl was enhanced in AuNT water. As shown in a previous study^38^, the SERS spectrum of polypyrrole (PPy) deposited on electrochemically roughened Au with a predominant (111) orientation exhibited a higher intensity and better resolution than that deposited on electrochemically roughened Au with a predominant (220) orientation. The result was ascribed to the significant appearance of an anodic peak at ca. 0.1~0.8 V vs. Ag/AgCl on an Au substrate with a predominant (111) orientation (Fig. 2b,c), but it disappeared on the Au substrate with a predominant (220) orientation (Fig. S2). Namely, the higher this anodic peak was, the stronger the SERS signal of PPy was obtained. Furthermore, this anodic peak initially showed up on the 5th scan. Figure 2b based on the DI water-system displays a similar phenomenon to that of a previous study^38^. As shown in Fig. 2c based on the AuNT water-system, it notably demonstrates a positive effect of increasing the anodic peak, which was expected with the correspondingly increased SERS enhancement. On the 25^th^ scan, the anodic peak current had increased from 3.45 to 4.18 mA (an increase of ~21%) when using an AuNT water-based solution instead of the conventional DI water-based solution. On the other hand, cathodic redeposition of Au ions on the same Au substrate with a predominant (220) orientation was easier in the AuNT water-based solution than in the DI water-based solution, which reflects the significantly enhanced peak current, as shown in Fig. S2. In addition, further inductively coupled plasma-mass spectrometer (ICP-MS) analyses indicated that the concentration of dissolved Au ions from the Au substrate (0.238 cm^2^) in 0.1 N NaCl (40 mL) after 500 scans had increased from 7.64 to 11.8 ppm (an increase of ~54%) when using the AuNT water-based solution instead of the DI water-based solution. These interesting electrochemical benefits based on AuNT water have not previously been reported in the literature.

Furthermore, similar experiments were performed in an electrolyte-free condition in DI water and in AuNt water using the same Au substrate, as shown in Fig. 2d. Based on results discussed in Fig. 2a, the triangular voltammetric curve in non-conductive DI water without the addition of electrolytes was featureless; while the triangular voltammetric curve in slightly conductive AuNT water without the addition of electrolytes was also featureless, but the recorded low current was markedly enlarged. Solid water (ice) has a nearly perfect tetrahedral symmetry around each water molecule; while liquid water deviates from the perfect tetrahedral symmetry of an HB network due to defects, thereby allowing it to play a central role in a vast number of chemical processes^9^,^32^,^39^,^40^,^41^, as well as in biological processes^42^. All of these liquid water-involving processes respond to liquid water’s activity from its available water molecules, not fully HB ones. Therefore, the created AuNT water with reduced HBs was intrinsically active, as demonstrated by the effective promotion of solubility, ionic conductivity, and electrochemical reactivity in AuNT water. These findings suggest that AuNT water is a promising innovative solvent for promoting water’s fundamental activities.

As reported in the literature, polyphenols^43^ and 2,3,5,4′-tetrahydroxystilbene-2-O-beta-d-glucoside (THSG)^44^ are antioxidative components of tea and PM, respectively. They are known to possess many health benefits. Therefore, further experiments were performed to examine if these healthy components could be more effectively extracted using high-energy AuNT water. Epigallocatechin gallate (EGCG) in polyphenols is the major active component of the health benefits of tea. Correspondingly, 2,2-diphenyl-1-picrylhydrazyl (DPPH) is a stable free radical, which has generally been employed to examine the free radical-scavenging capacity of various chemicals^45^, including EGCG^46^. Therefore, the health benefits of scavenging DPPH by tea solutions extracted using AuNT water at room temperature were examined. Figure 3a demonstrates the scavenging abilities on DPPH free radicals of AuNT water-extracted tea solutions. Figure S3 demonstrates the corresponding electron spin resonance (ESR) spectra. The main ESR signal shown at ca. 3482 G is a characteristic of DPPH radicals^46^. Encouragingly, DPPH radicals were significantly reduced in the tea solutions extracted using AuNT water, compared to those extracted using DI water. The corresponding ESR intensities decreased by ~20% and ~17% for green tea (non-oxidized tea) and Pu-er tea (post-oxidized tea), respectively, based on AuNT water. Figure 3b demonstrates the corresponding EGCG contents in tea solutions extracted using different waters. Consistently, EGCG contents were significantly higher in tea solutions extracted using AuNT water, compared to those extracted using DI water. The corresponding EGCG contents increased by ~21% and ~10% for green tea and Pu-er tea, respectively, based on AuNT water. To the best of our knowledge, this increase in EGCG contents of tea solutions extracted using high-energy AuNT liquid water at room temperature is the first report in the literature. EGCG is classified as a polyphenol with characteristic multi-OH groups, which can form intermolecular HBs with water molecules. As recently reported^47^, the electrochemical properties of phenols and quinones in organic solvents are strongly influenced by HBs of water. It was also reported by our group^20^ that compared to DI water, there is more ‘free water’ in AuNT water with weaker HBs, which can strongly interact with hydroquinone (HQ) by HBs. Therefore, EGCG was more effectively extracted from teas utilizing AuNT water with more free water molecules that could form effective intermolecular HBs with polyphenols.

Figure 4 demonstrates THSG contents from PM, which were extracted with ethanol (EtOH) solutions (60% EtOH in DI water and AuNT water) at room temperature, in crude extraction solutions and further lyophilized powders. Figure 4a shows THSG contents of crude extraction solutions. Interestingly, the THSG content was significantly higher in the crude extract based on AuNT water (28.11 ± 1.33 mg g^−1^), compared to that based on DI water (25.19 ± 1.20 mg g^−1^). The corresponding THSG content significantly increased by ~12% (*p* < 0.05; this *p* value means that the deviation probability of the experimental data was <0.05, which indicates that the experimental result was significant) for PM extracted using an AuNT water-based solvent. Similarly and more significantly in statistics, as shown in Fig. 4b based on lyophilized powder dissolved in DI water, the THSG content was significantly higher in the lyophilized sample based on AuNT water (109.6 ± 0.2 mg g^−1^), compared to that based on DI water (131.3 ± 0.2 mg g^−1^). The corresponding THSG content increased by ~20% (*p* < 0.001), which was higher than ~12% for the crude extract, for PM extracted using the AuNT water-based solvent. This result suggests that a trace of AuNT water in lyophilized samples can suppress oxidation of THSG, as revealed from the antioxidant capacity of AuNT water^20^. Moreover, total extraction yields based on lyophilized samples (See SI) were 14.3% and 16.3% for the DI water- and AuNT water-based solvents, respectively. This is an increase of ~14% in the extraction efficiency using the AuNT water-based solvent. THSG in PM is also a kind of polyphenol with multiple OH groups, which can form intermolecular HBs with water molecules. Therefore, AuNT water with more free water molecules, which can form more intermolecular HBs, is responsible for the more-effective extraction of THSG from PM.

As discussed above, AuNT water’s weak HBs are responsible for its better fundamental abilities. In addition, this effect on the corresponding evaporation rate was examined in ambient laboratory air. As shown in Fig. 5a, in the first hour, the magnitude of the evaporation rate of AuNT water was markedly higher by ca. 32%, compared to that of DI water. Finally, in the fourth hour, this increase of evaporation rate was greatly reduced to ca. 5.6%. These results suggest that active water molecules in the as-prepared AuNT water quickly evaporated in the first hour, after which the originally distinct property distinguishable from DI water was greatly reduced in this experiment-induced aged AuNT water. Figure 5b demonstrates the evaporation rates per hour of different aqueous solutions of alkali metal-chloride salts. Based on the colligative properties of ionic solutions, the vapor pressure of ionic solutions should be lower than that of the pure solvent. Thus the evaporation rate of the alkali metal-chloride salt-containing aqueous solution was lower than that of pure water, as can be seen by comparing Fig. 5b,a. For example, in a 0.9 wt% NaCl solution (normal saline solution) based on DI water, the magnitude of the evaporation rate in the first hour decreased by ca. 5.2% compared to that of pure DI water. This decrease was ca. 13% in magnitude based on AuNT water. Similarly, for a 0.9 wt% LiCl solution based on DI water, the magnitude of the evaporation rate in the first hour was slightly lower by ca. 2.9%, compared to that of pure DI water. This decrease was greatly enhanced to ca. 27% in magnitude based on AuNT water. However, for 0.9 wt% KCl solutions based on DI water and AuNT water, the decreases in evaporation rates compared to those of individual pure water were comparable, with decreases of ca. 12% and 8.7% in magnitude for DI water-based and AuNT water-based systems. These phenomena, correlated with their differently increased saturated solubilities in AuNT water, are interesting and are discussed below. Nevertheless, AuNT water-based systems also led to correspondingly faster evaporation rates in 0.9 wt% alkali metal-chloride salt solutions, compared to those of DI water-based systems. In the first hour (left plot), magnitudes of evaporation rates of LiCl, NaCl, and KCl in AuNT waters were respectively higher by ca. 0.0%, 21%, and 38%, compared to those of LiCl, NaCl, and KCl in DI waters. Interestingly, this increasing tendency was inversely proportional to the increasing tendency of the solubilities of different salts in AuNT water, compared to DI water, as illustrated in Fig. 1a. These phenomena resulted because most of the active water molecules in AuNT water are consumed in dissolving LiCl to greatly reduce its hydration energy (its increased saturated solubility in the AuNT water was the largest). Thus, the originally distinct activity distinguishable from DI water was greatly reduced, which reflects the equal evaporation rate of LiCl solutions in both DI water and AuNT water. This effect also resulted in a great decrease in the evaporation rate of ca. 27% in magnitude for the 0.9 wt% LiCl solution based on AuNT water, compared to that of pure AuNT water. Also, the average evaporation rates per hour for the first 4 hours (right plot) of LiCl, NaCl, and KCl in AuNT waters were respectively higher by ca. 0.38%, 4.7%, and 7.5%, compared to those of LiCl, NaCl, and KCl in DI waters. Conclusively, the distinct properties of AuNT water led to correspondingly faster evaporation rates for both itself and its ionic solutions. The energetic AuNT water created from HET can released its reserved energy in dissolving salts more effectively. Encouragingly, the remaining reserve energy in AuNT water, compared to DI water, was also available, which can lead to faster evaporation rates of ionic solutions. This energy-progress process of the utilized AuNT water is illustrated in Fig. 5c. In addition, this innovative idea of validating the reserve energy in AuNT water is exhibited in Figure S2 regarding the ORC procedure for roughening the same Au substrate (in a predominant (220) orientation) in different waters containing different 0.1 N alkali metal-chloride salts. As demonstrated in Figure S2, increased cathodic redeposition currents on the 25^th^ scans of KCl, NaCl, and LiCl in AuNT waters were respectively increased by ca. 44%, 13%, and 2.0%, compared to those of LiCl, NaCl, and KCl in DI waters. This increasing tendency was inversely proportional to the increasing trends in solubility of the different salts in AuNT water, compared to DI water, as discussed in Fig. 1a.

We successfully utilized plasmon-induced high-energy AuNT liquid water to promote water’s fundamental activities of solubility, ionic conductivity, and extraction at room temperature. In addition, the innovative idea of validating the reserve energy in AuNT water was proposed. The solubility of alkali metal-chloride salts in AuNT water was significantly increased by increasing the corresponding hydration energy in the solution process. The ionic conductivity of NaCl in AuNT water was also markedly increased at extremely low concentrations. In addition, EGCG and THSG were more effectively extracted from teas and PM, respectively, by utilizing AuNT water with more free water molecules than was possible from effectively intermolecular HBs with polyphenols. Moreover, the distinct property of AuNT water led to correspondingly fast evaporation rates for both itself and its ionic solutions. These encouraging findings, which are the first shown in the literature, demonstrate that high-energy water has emerged as a promising innovative solvent for promoting water’s fundamental activities via effective energy transfer.

## Additional Information

**How to cite this article**: Yang, C.-P. *et al.* Effective Energy Transfer via Plasmon-Activated High-Energy Water Promotes Its Fundamental Activities of Solubility, Ionic Conductivity, and Extraction at Room Temperature. *Sci. Rep.* **5**, 18152; doi: 10.1038/srep18152 (2015).

## Supplementary Material

Supplementary Information

## Figures and Tables

**Figure 1:**
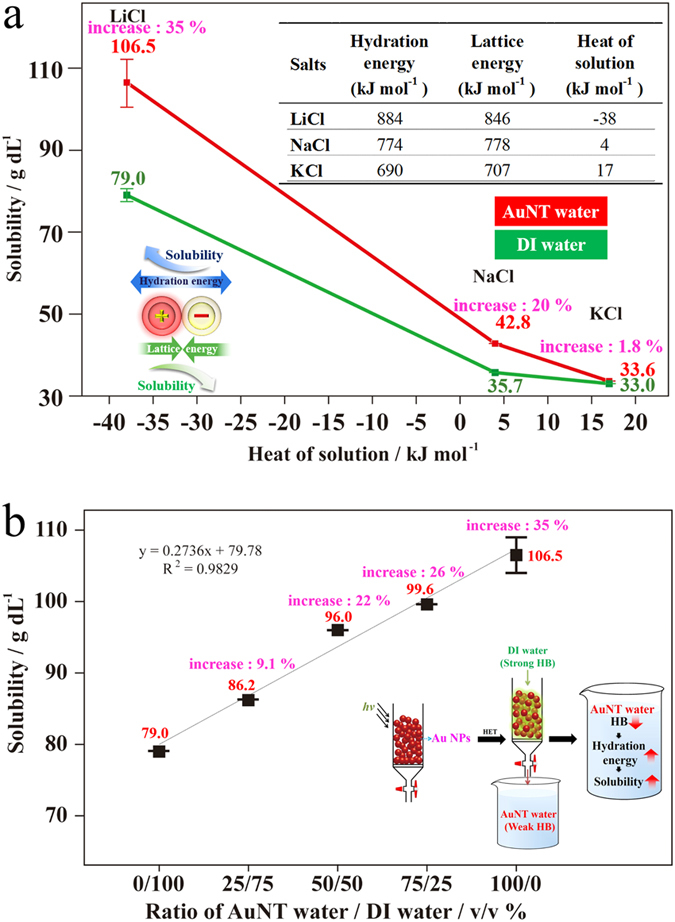
Solubilities of alkali metal-chloride salts in deionized (DI) water and in gold nanoparticle-treated (AuNT) water at room temperature. (**a**) Dependence of an increase in salt solubility in AuNT water, compared to salt dissolved in DI water, on its corresponding heat of solution; inset demonstrates the heats of solutions, and their individual lattice and hydration energies of the corresponding salts in conventional DI water. (**b**) Solubility of LiCl in water composed of different ratios of AuNT water in DI and AuNT waters.

**Figure 2:**
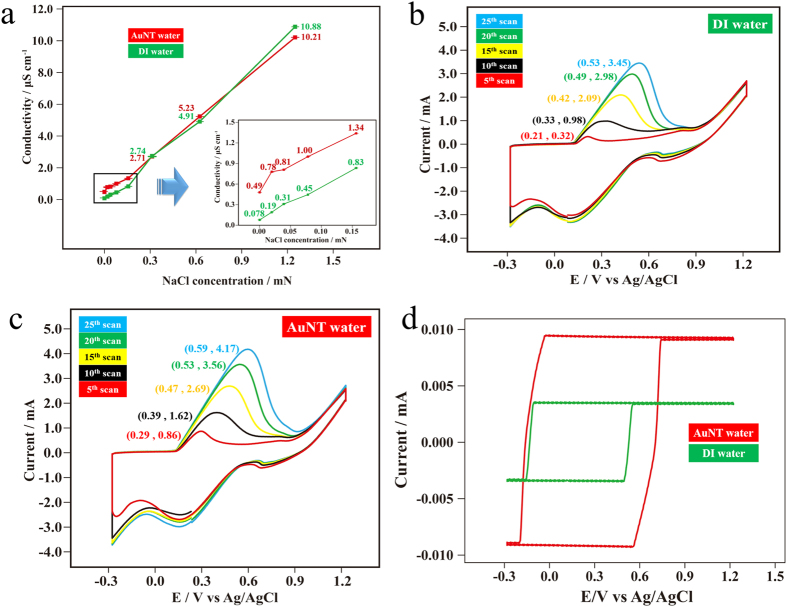
Ionic conductivities of NaCl in different waters at room temperature and their effects on the corresponding electrochemical reactivities. (**a**) Dependence of an increase in the ionic conductivity on NaCl in gold nanoparticle-treated (AuNT) water, compared to NaCl in DI water, on the corresponding concentration of NaCl. (**b**) Cyclic voltammograms (CVs) showing different scans in oxidation-reduction cycle (ORC) treatments with a roughened Au electrode in 0.1 N NaCl in DI water. The ORC condition for the Au electrode was −0.28 to +1.22 V vs. Ag/AgCl at 500 mV/s with respective durations at the cathodic and anodic vertices of 10 and 5 s. (**c**) CVs showing different scans in ORC treatments with a roughened Au electrode in 0.1 N NaCl in AuNT water. The ORC condition and the Au electrode were the same as those used in DI water. (**d**) CVs showing the 25th scans in ORC treatments with the same roughened Au electrode in DI water and in AuNT water without electrolytes.

**Figure 3:**
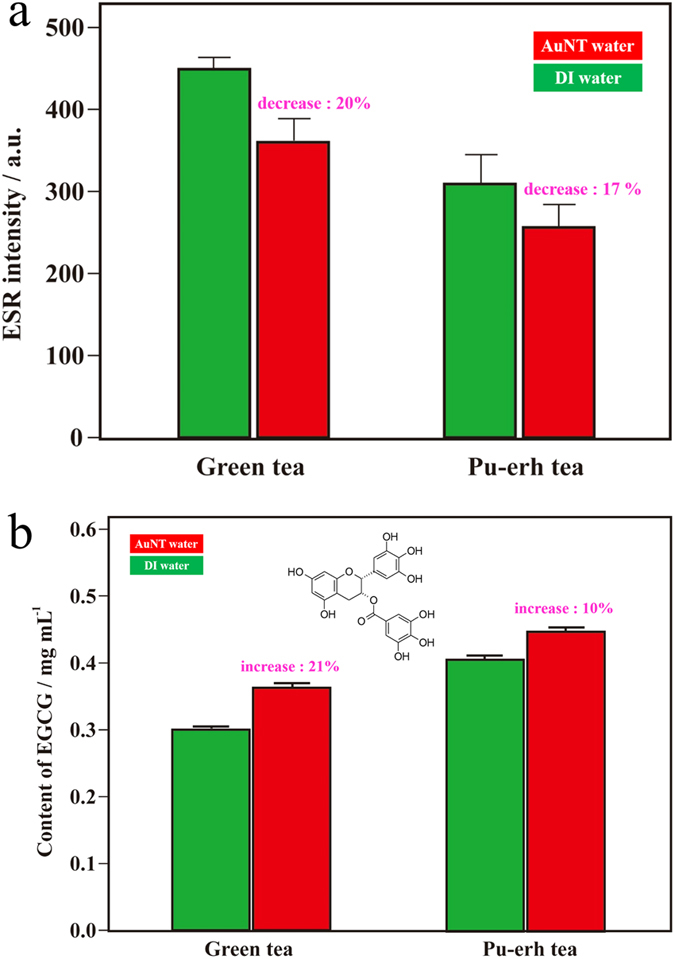
Antioxidative abilities of teas and coffees extracted using deionized (DI) water and gold nanoparticle-treated (AuNT) water at room temperature (a 1-g sample was dissolved in 50 g of water). (**a**) ESR spectra of 2,2-diphenyl-1-picrylhydrazyl (DPPH) free radicals based on extracted green teas. The inset demonstrates the epigallocatechin gallate (EGCG; the major polyphenol) contents in green tea and Pu-er tea extracted using DI water and AuNT water at room temperature. (**b**) UV-vis spectra showing the relative concentrations of caffeic acids in the extracted coffees.

**Figure 4:**
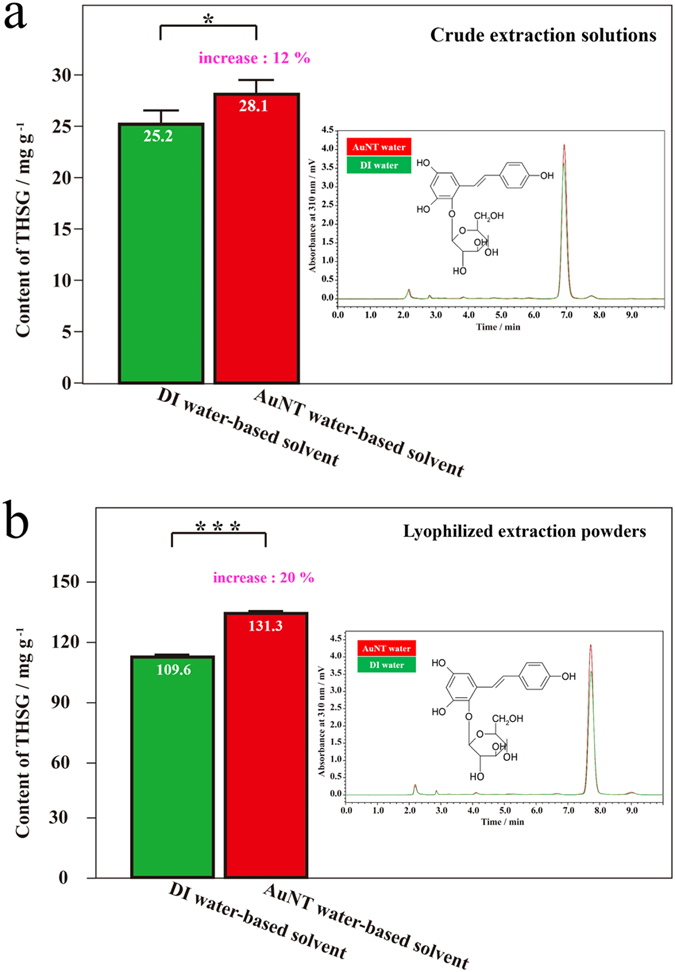
Contents of 2,3,5,4′-tetrahydroxystilbene-2-O-beta-d-glucoside (THSG) in different types of extracts from *Polygonum multiflorum* (PM) extracted using different water-based solvents. (**a**) In crude extraction solutions; the inset demonstrates the corresponding HPLC analyses to calculate the contents of THSG in DI water-based and AuNT water-based solvents; *p < 0.05. (**b**) In extraction powder; the inset demonstrates the corresponding HPLC analyses to calculate the contents of THSG from DI water-based and AuNT water-based solvents; ***p < 0.001.

**Figure 5:**
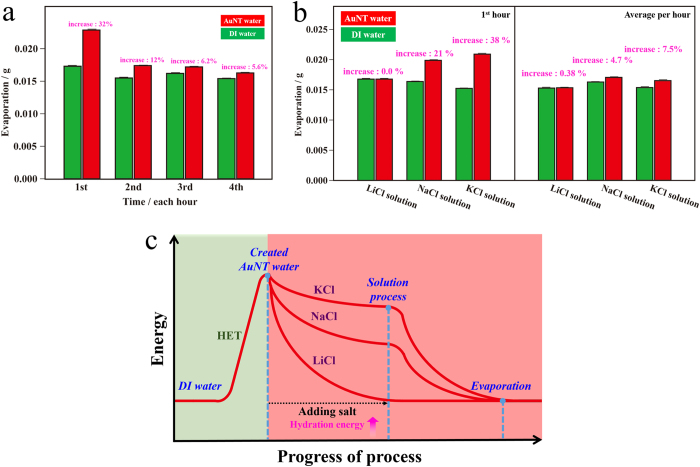
Evaporation rates of deionized (DI) water, gold nanoparticle-treated (AuNT) water and alkali metal-chloride salt-containing DI water and AuNT water at 1 atm and room temperature with 52% relative humidity (RH). (**a**) Evaporation rates per hour of DI water and AuNT water with respect to the passed time. (**b**) Evaporation rates per hour based on the first hour (left plot) and the first 4 hours (right plot) of 0.9 wt% alkali metal-chloride salt-containing DI water and AuNT water. (**c**) Schematic descriptions of energy-progress of process curve of the process curve of activated AuNT water in effective energy transfer for dissolution and evaporation processes.

## References

[b1] Panman M. R. *et al.* Water lubricates hydrogen-bonded molecular machines. Nat. Chem. 5, 929–934 (2013). 24153370 10.1038/nchem.1744

[b2] Samanta A. K., Czakó G., Wang Y., Mancini J. S., Bowman J. M. & Reisler H. Experimental and theoretical investigations of energy transfer and hydrogen-bond breaking in small water and HCl clusters. Acc. Chem. Res. 47, 2700–2709 (2014). 25072730 10.1021/ar500213q

[b3] Kou J., Lu H., Wu F., Fan J. & Yao J. Electricity resonance-induced fast transport of water through nanochannels. Nano Lett. 14, 4931–49367 (2014). 25019561 10.1021/nl500664y

[b4] David J. G., Gierszal K. P., Wang P. & Ben-Amotz D. Water structural transformation at molecular hydrophobic interfaces. Nature 491, 582–585 (2012). 23172216 10.1038/nature11570

[b5] Siretanu I., Chapel J. P. & Drummond C. Water-ions induced nanostructuration of hydrophobic polymer surfaces. Science 292, 908–912 (2001). 21434645 10.1021/nn103564e

[b6] Kou J., Lu H., Wu F., Fan J. & Yao J. Electricity resonance-induced fast transport of water through nanochannels. ACS Nano 4, 2939–2947 (2011). 10.1021/nl500664y 25019561

[b7] Velasco-Velez J. J. *et al.* The structure of interfacial water on gold electrodes studied by x-ray absorption spectroscopy. Science 346, 831–834 (2014). 25342657 10.1126/science.1259437

[b8] Murata K. *et al.* Structural determinants of water permeation through aquaporin-1. Nature 407, 599–605 (2000). 11034202 10.1038/35036519

[b9] Shen J. Y. *et al.* Probing water micro-solvation in proteins by water catalyzed proton-transfer tautomerism. Nat. Commun. 4, 2611 (2013). 24177573 10.1038/ncomms3611

[b10] Schuck P. J. Hot electrons go through the barrier. Nat. Nanotech. 8, 799–800 (2013). 10.1038/nnano.2013.228 24141537

[b11] Giugni1 A. *et al.* Hot-electron nanoscopy using adiabatic compression of surface plasmons. Nat. Nanotech. 8, 845–852 (2013). 10.1038/nnano.2013.207 24141538

[b12] Sundararaman R., Narang P., Jermyn A. S., Goddard W. A. III & Atwater H. A. Theoretical predictions for hot-carrier generation from surface plasmon decay. Nat. Commun. 5, 5788 (2014). 25511713 10.1038/ncomms6788 PMC4284641

[b13] Mukherjee S. *et al.* Hot electrons do the impossible: plasmon-induced dissociation of H_2_ on Au. Nano Lett. 13, 240–247 (2013). 23194158 10.1021/nl303940z

[b14] Sil D. *et al.* Seeing is believing: hot electron based gold nanoplasmonic optical hydrogen sensor. ACS Nano 8, 7755–7762 (2014). 25072929 10.1021/nn500765t

[b15] Qian K. *et al.* Surface plasmon-driven water reduction: gold nanoparticle size matters. J. Am. Chem. Soc. 136, 9842–9845 (2014). 24972055 10.1021/ja504097v

[b16] DuChene J. S. *et al.* Prolonged hoe electron dynamics in plasmonic-metal/semiconductor heterostructures with implications for solar photocatalysis. Angew. Chem. Int. Ed. 53, 7887–7891 (2014). 10.1002/anie.201404259 24920227

[b17] Sheldon M. T., van de Groep J., Brown A. M., Polman A. & Atwater H. A. Plasmoelectric potentials in metal nnaostructures. Science 346, 828–831 (2014). 25395532 10.1126/science.1258405

[b18] Kang Y. *et al.* Plasmonic hoe electron induced structural phase transition in a MoS_2_ monolayer. Adv. Mater. 26, 6467–6471 (2014). 25100132 10.1002/adma.201401802

[b19] Pescaglini A. *et al.* Hot-electron injection in Au nanorod–ZnO nanowire hybrid device for near-infrared photodetection. Nano Lett. 14, 6202–6209 (2014). 25313827 10.1021/nl5024854

[b20] Chen H. C. *et al.* Active and stable liquid water innovatively prepared using resonantly illuminated gold nanoparticles. ACS Nano 8, 2704–2713 (2014). 24533852 10.1021/nn406403c

[b21] Chen H. C. *et al.* Innovative strategy with potential to increase hemodialysis efficiency and safety. Sci. Rep. 4, 4425 (2014). 24651843 10.1038/srep04425 PMC3961733

[b22] Woutersen S. & Bakker H. J. Resonant intermolecular transfer of vibrational energy in liquid water. Nature 402, 507–509 (1999).

[b23] Concepcion J. J., House R. L., Papanikolas J. M. & Meyer T. J. Chemical approaches to artificial photosynthesis. Proc. Natl. Acad. Sci. USA. 109, 15560–15564 (2012). 23019352 10.1073/pnas.1212254109 PMC3465377

[b24] Prier C. K., Rankic D. A. & MacMillan D. W. C. Visible light photoredox catalysis with transition metal complexes: applications in organic synthesis. Chem. Rev. 113, 5322–5363 (2013). 23509883 10.1021/cr300503r PMC4028850

[b25] Ghosh I., Ghosh T., Bardagi J. I. & Konig B. Reduction of aryl halides by consecutive visible light-induced electron transfer processes. Science 346, 725–728 (2014). 25378618 10.1126/science.1258232

[b26] Hammarström L. Accumulative charge separation for solar fuels production: coupling light-induced single electron transfer to multielectron catalysis. Acc. Chem. Res. 48, 840–850 (2015). 25675365 10.1021/ar500386x

[b27] van den Berg C. & Bruin S. Water activity and its estimation in food systems: theoretical aspects. In: Rockland L. B.; Stewart G. F. (Eds). Water activity: influences on food quality. New York: Academic Press, 1981.

[b28] Baer C. & Adamus S. M. The solubility of ionic solids and molecular liquids J. Chem. Educ. 76, 1540–1541 (1999).

[b29] Cogoni G., Baratti R. & Romagnoli J. A. On the influence of hydrogen bond interactions in isothermal and nonisothermal antisolvent crystallization processes Ind. Eng. Chem. Res. 52, 9612–9619 (2013).

[b30] Biedermann F., Nau W. M. & Schneider H. J. The hydrophobic effect revisited-studies with supramolecular complexes imply high-energy water as a nonconvalent driving force. Angew. Chem. Int. Ed. 53, 2–16 (2014). 10.1002/anie.201310958 25070083

[b31] Pérez C. *et al.* Hydrogen bond cooperativity and the three-dimensional structures of water nonamers and decamers. Angew. Chem. Int. Ed. 53, 14368–14372 (2014). 10.1002/anie.201407447 25348841

[b32] Mifsud M. *et al.* Photobiocatalytic chemistry of oxidoreductases using water as the electron donor. Nat. Commun. 5, 3145 (2014). 24473192 10.1038/ncomms4145

[b33] Langton M. J., Robinson S. W., Marques I., Felix V. & Beer P. D. Halogen bonding in water results in enhanced anion recognition in acyclic and rotaxane hosts. Nat. Chem. 6, 1039–1043 (2014). 25411880 10.1038/nchem.2111

[b34] Piatkowski L., Zhang Z., Backus E. H. G., Bakker H. & Bonn M. Extreme surface propensity of halide in water. Nat. Commun. 5, 4083 (2014). 24899106 10.1038/ncomms5083

[b35] David J. G., Rankin B. M., Gierszal K. P. & Ben-Amotz D. On the cooperative formation of non-hydrogen-bonded water at molecular hydrophobic interfaces. Nat. Chem. 5, 796–802 (2013). 23965683 10.1038/nchem.1716

[b36] Liu Y. C., Hsu T. C. & Tsai J. F. Thermal stability of electrochemically prepared surface-enhanced raman scattering-active metals substrates. J. Phys. Chem. C 111, 10570–10574 (2007).

[b37] Wang C. C. Surfaced-enhanced raman scattering-active substrates prepared through a combination of argon plasma and electrochemical techniques. J. Phys. Chem. C 112, 5573–5578 (2008).

[b38] Liu Y. C. & Jang L. Y. Relationship between crystalline orientations of gold and surface-enhanced raman scattering spectroscopy of polypyrrole and mechanism of roughening procedure on gold via cyclic voltammetry. J. Phys. Chem. B 106, 6748–6753 (2002).

[b39] Merte L. R. *et al.* Water-mediated proton hopping on an iron oxide surface. Science 336, 889–893 (2012). 22605771 10.1126/science.1219468

[b40] Rivard U., Thomas V., Brufacs A., Siwck B. & Lftimie R. Donor-bridge-acceptor proton transfer in aqueous solution. J. Phys. Chem. Lett. 5, 3200–3205 (2014). 26276332 10.1021/jz501378d

[b41] Buszek R. J., Barker J. R. & Francisco J. S. Water effect on the OH + HCl reaction. J. Phys. Chem. A 116, 4712–4719 (2012). 22563978 10.1021/jp3025107

[b42] Kou J., Lu H., Wu F., Fan J. & Yao J. Electricity resonance-induced fast transport of water through nanochannels. Nano Lett. 14, 4931–4936 (2014). 25019561 10.1021/nl500664y

[b43] Neshchadin D., Batchelor S. N., Bilkis I. & Gescheidt G. Short-lived phenoxyl radicals formed from green-tea polyphenols and highly reactive oxygen species: an investigation by time-resolved EPR spectroscopy. Angew. Chem. Int. Ed. 53, 13288–13292 (2014). 10.1002/anie.201407995 25345684

[b44] Lv L. *et al.* Stilbene glucoside from polygonum multiflorum thumb.: a novel natural inhibitor of advanced glycation end product formation by trapping of methylglyoxal. J. Agric. Food Chem. 58, 2239–2245 (2010). 20104848 10.1021/jf904122q

[b45] Chen Y., Wang M., Rosen R. T. & Ho C. T. 2,2-diphenyl-1-picrylhydrazyl radical-scavenging active components from *polygonum multiflorum* thumb. J. Agric. Food Chem. 47, 2226–2228 (1999). 10794614 10.1021/jf990092f

[b46] Zhong Y. & Shahidi F. Lipophilized epigallocatechin gallate (EGCG) derivatived as novel antioxidants. J. Agric. Food Chem. 59, 6526–6533 (2011). 21526762 10.1021/jf201050j

[b47] Tessensohn M. E., Hirao H. & Webster R. D. Electrochemical properties of phenols and quinones in organic solvents are strongly influenced by hydrogen-bonding with water. J. Phys. Chem. C 117, 1081–1090 (2013).

