# Supplementary material for: Effective Energy Transfer via Plasmon-Activated High-Energy Water Promotes Its Fundamental Activities of Solubility, Ionic Conductivity, and Extraction at Room Temperature
Source: Sci Rep. 2015 Dec 10;5:18152. doi: 10.1038/srep18152 (PMC4674797; doi:10.1038/srep18152)
Supplement: Supplementary Information [file srep18152-s1.doc]

Supplementary Information

**Effective Energy Transfer via Plasmon-Activated High-Energy Water Promotes Its Fundamental Activities of Solubility, Ionic Conductivity, and Extraction at Room Temperature**

Chih-Ping Yang^1^, Hsiao-Chien Chen^2^, Ching-Chiung Wang^3^, Po-Wei Tsai^3^, Chia-Wen Ho^4^ & Yu-Chuan Liu^2,5,*^

^1^Graduate Institute of Medical Science, College of Medicine, Taipei Medical University, 250 Wuxing St., Taipei 11031, Taiwan

^2^Department of Biochemistry and Molecular Cell Biology, School of Medicine, College of Medicine, Taipei Medical University, 250 Wuxing St., Taipei 11031, Taiwan

^3^School of Pharmacy, College of Pharmacy, Taipei Medical University, 250 Wuxing St., Taipei 11031, Taiwan

^4^Center for Cancer Research, Taipei Medical University, 250 Wuxing St., Taipei 11031, Taiwan

^5^Biomedical Mass Imaging Research Center, Taipei Medical University, 250 Wuxing St., Taipei 11031, Taiwan

^*^ Corresponding author

Tel: 886-2-27361661 ext 3155; Fax: 886-2-27356689; E-mail: [liuyc@tmu.edu.tw](mailto:liuyc@tmu.edu.tw)

**Supplementary Materials**

1. Chemicals and materials

Electrolytes of LiCl, NaCl, and KCl and reagents of 2,2-diphenyl-1-picrylhydrazyl (DPPH) and epigallocatechin gallate (EGCG) were purchased from Sigma-Aldrich Organics. Radix *Polygonum multiflorum* (PM) was purchased from pharmacies in Sichuan Province, China. 2,3,5,4'-Tetrahydroxystilbene-2-O-beta-d-glucoside (THSG, with a purity of >97%) was purchased from the National Institute for the Control of Pharmaceutical and Biological Products, China. All of the reagents were used as received without further purification. All of the solutions were prepared using deionized (DI) 18.2 MΩ cm water from a Milli-Q system. All of the experiments were performed in an air-conditioned room at ca. 23 °C. The water temperature was ca. 22.9 °C.

**Supplementary Methods**

1. Preparation of AuNT water

AuNT water was prepared by a previously described method^20^. Typically, DI water (pH 6.95, T = 22.9 °C) was passed through a glass tube filled with Au NP-adsorbed ceramic particles under resonant illumination of green light-emitting diodes (LEDs, with wavelength maxima centered at 530 nm). Then AuNT water (pH 6.96, T = 22.9 °C) was collected in glass sample bottles for subsequent measurements within 2 h.

2. Saturated solubility of alkali metal-chloride salts in water at room temperature

The solubility of alkali metal-chloride salts in water was obtained by dissolving excess salt in 20 mL of water under stirring for 30 min. Then the solution was placed without stirring for another 30 min. Subsequently, five samples of 1 mL of the clear salt-saturated solutions were weighed. The saturated solubility of NaCl based on 1 dL of water was calculated by utilizing the known densities of 2.068 and 1 g cm^-3^ for LiCl and water, respectively. Similarly, densities of 2.165 and 1.987 g cm^-3^ were used for NaCl and KCl, respectively, in the calculations.

3. Ionic conductivity of NaCl in water at room temperature

Ionic conductivities of pure water and an NaCl-containing aqueous solution were measured using an ionic conductivity meter (model: Cond 330i, WTW, Germany). Replicate measurements based on five similar samples were performed.

4. Evaporation rate of water at room temperature into ambient laboratory air

Samples of 20 mL of water (or aqueous solutions) were added to open glass sample bottles (20 mL), which were placed on a platform of an orbital shaker, operating at 150 rpm. The weight of each glass sample bottle, containing water or solution, was measured every hour for 4 h to determine the evaporating mass (g) of water per hour. In these experiments, the relative humidity (RH) was ca. 52%. Replicate measurements based on five similar samples were performed.

5. Extraction of tea and coffee using water at room temperature

First, stock samples of dry teas and coffees were evenly ground. The powder sizes were ca. 200 µm for both teas and coffees (Figure S4). Then 1 g of tea (10 g of coffee) powder was individually added to 50 mL of DI water and to 50 mL of AuNT water (120 mL of water for coffee). Subsequently, sample-containing solutions were extracted under violent stirring for 10 min; then the solutions were allowed to stand without stirring for another 30 min. Finally, the upper sample-containing clear solutions were filtered using commercial filter paper (model: 5C/90 mm, pore size of 5 µm, Toyo Roshi Kaisha, Japan) for subsequent tests.

6. Extraction of pm by an ethanol aqueous solution

Processed powder (1 g) of PM. was extracted with 10-mL ethanol (EtOH) solutions (60% EtOH in DI water and AuNT water) at room temperature. All of the sample solutions were placed in an ultrasonic bath for 2 h, and then filtered. The obtained supernatants in DI water-based solutions and in AuNT water-based solutions were individually separated into two parts for subsequent analyses. One part of the supernatant was directly analyzed by high-performance liquid chromatography (HPLC; model: LC-2010C HT, Shimadzu, Japan) to obtaining the THSG concentration in this crude extract based on a standard calibration curve of peak area vs. concentration (Figure S5). The other part of the supernatant was further lyophilized to from a powder. Then the PM-containing powder was dissolved in DI water (10 mg mL^-1^) and analyzed by HPLC to obtain the THSG concentration of the powder. In HPLC experiments, a 10-µL sample was injected into the oven (40 °C) at a flow rate of 1 mL min^-1^, and a Purospher® STAR RP-18e reversed-phase column (5 μm, 4 mm i.d. × 250 mm, Merck, Germany) and UV detector at 310 nm were employed.

Also, the total extraction yield based on the lyophilized sample was calculated, as defined below:

Yield (%) = [extracted and lyophilized powders / initial powders of PM] × 100% (1)

7. Measurement of free radicals of DPPH by electron spin resonance (ESR) spectroscopy

In ESR measurements, a Bruker EMX ESR spectrometer was employed. ESR spectra were recorded at room temperature using a flat quartz cell designed for solutions. The dead time between sample preparation and the ESR analysis was exactly 10 min for experiments on DPPH free radicals after the last addition. Conditions of ESR spectrometry were as follows: 20 mW power at 9.78 GHz, with a scan range of 100 G and a receiver gain of 6.32 × 10^4^. Before the ESR analysis, the sample was prepared as described here.

DPPH was dissolved in methanol to prepare a 4-mM DPPH stock solution. A tea solution from the extraction using AuNT water (or DI water) was also prepared. Then the prepared DPPH solution and tea solution were mixed (100 μL each) in a microtube. The final concentration of DPPH in the solution was 2 mM. Exactly 10 min after mixing the DPPH and tea solutions, the ESR analysis was performed. To measure an ESR spectrum, a sample was scanned one time (for ca. 42 s).

8. Contents of EGCG in tea solutions

Total EGCG contents in tea solutions from extraction using AuNT water (or DI water) were measured by a method shown in the literature.^1^ Typically, an absorbance at 765 nm of the tea-dissolved solution was recorded on an enzyme-linked immunosorbent assay (ELISA) reader (BioTek, USA) to determine the concentration of EGCG in the tea solution based on a standard calibration curve of absorbance vs. concentration (Figure S6).


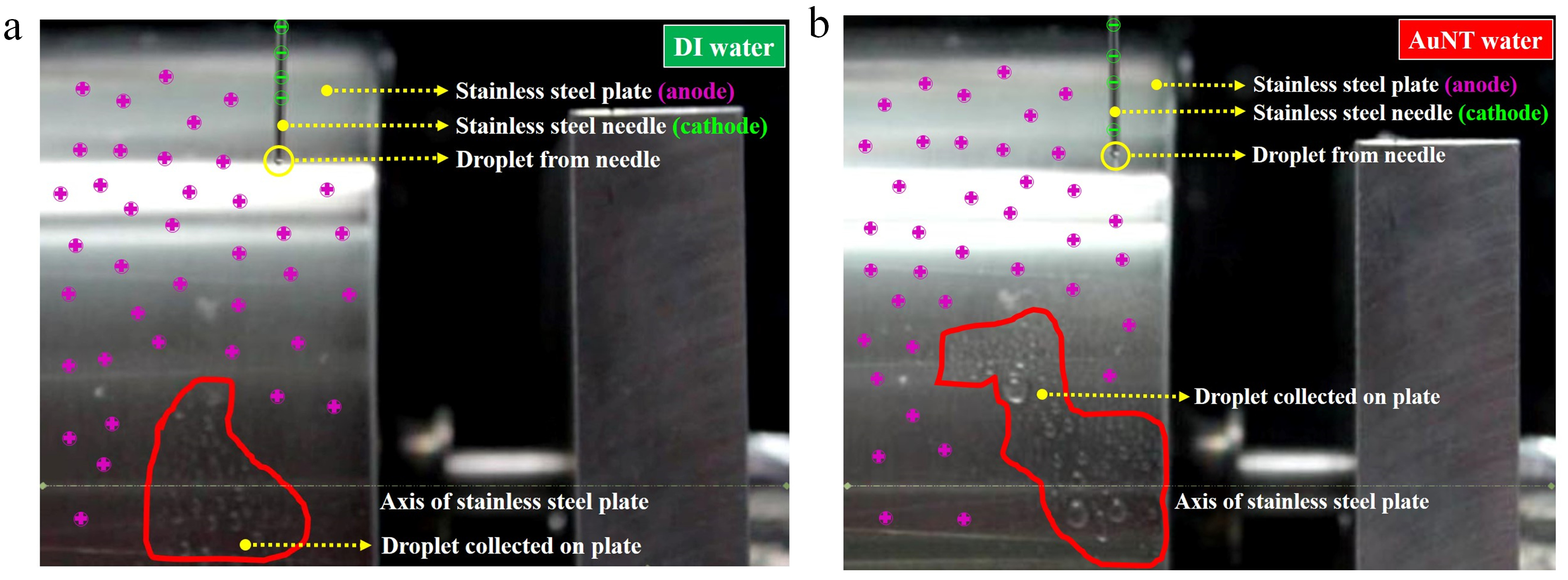


**Figure S1 │** Photos showing slanting droplets of deionized (DI) water (a) and gold nanoparticle-treated (AuNT) water (b), which were sprayed from a stainless steel needle (cathode) in an electron-spin module^2^ at 10 kV, toward a grounded stainless steel plate counter electrode (anode). In stationary electric fields, most of the droplets of AuNT water from the cathodic needle were collected above the central axis of the anodic plate; while most of the droplets of DI water were collected below the central axis of the anodic plate. Moreover, more droplets of AuNT water, compared to DI water, were observed on the anodic plate.


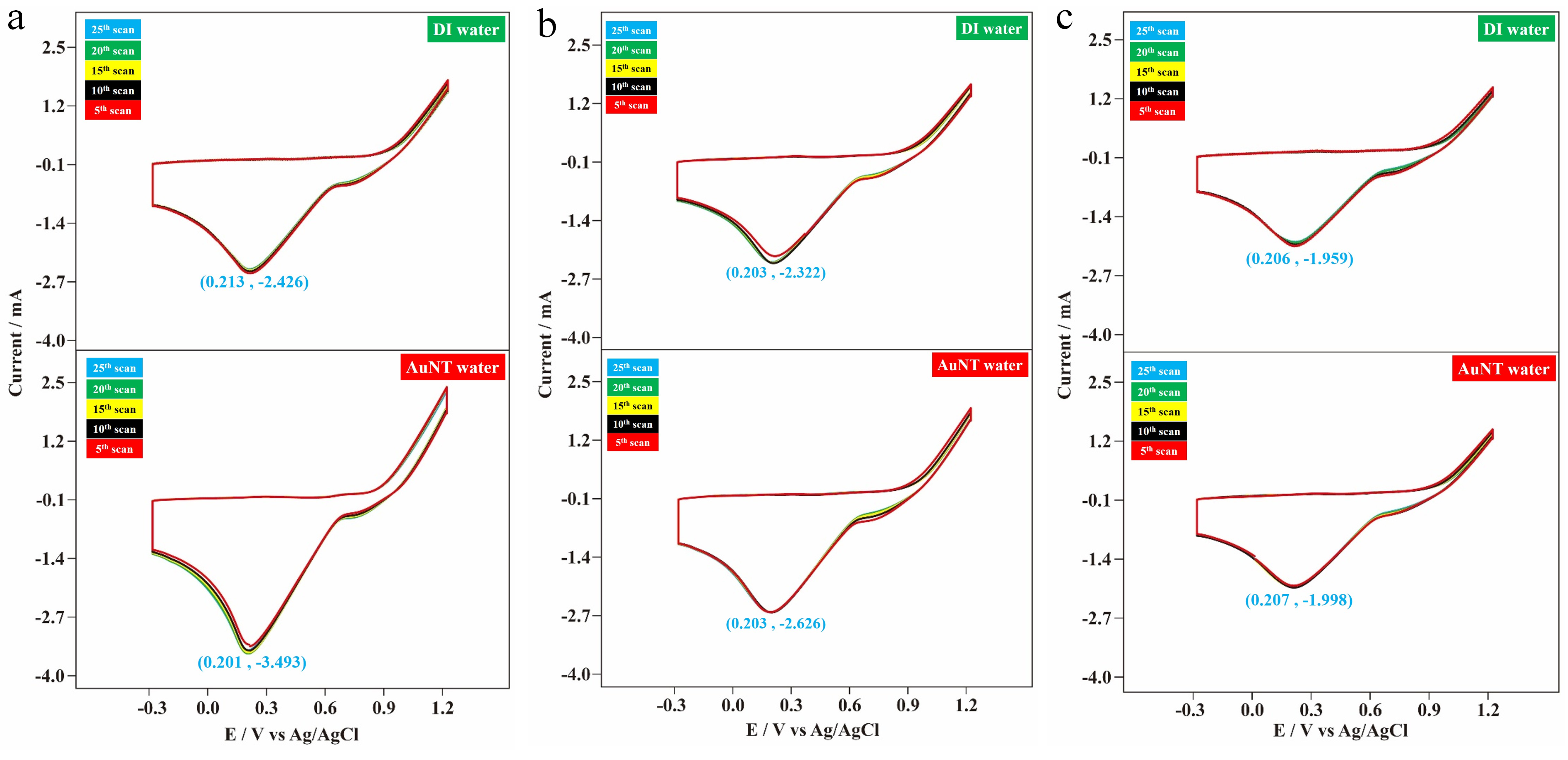


**Figure S2 │** Cyclic voltammograms (CVs) showing different scans in oxidation-reduction cycle (ORC) treatments for roughening the same Au electrode (in a predominant (220) orientation) in different waters containing different 0.1 N alkali metal-chloride salts. (a). In 0.1 N KCl; (b). In 0.1 N NaCl; (c). In 0.1 N LiCl.


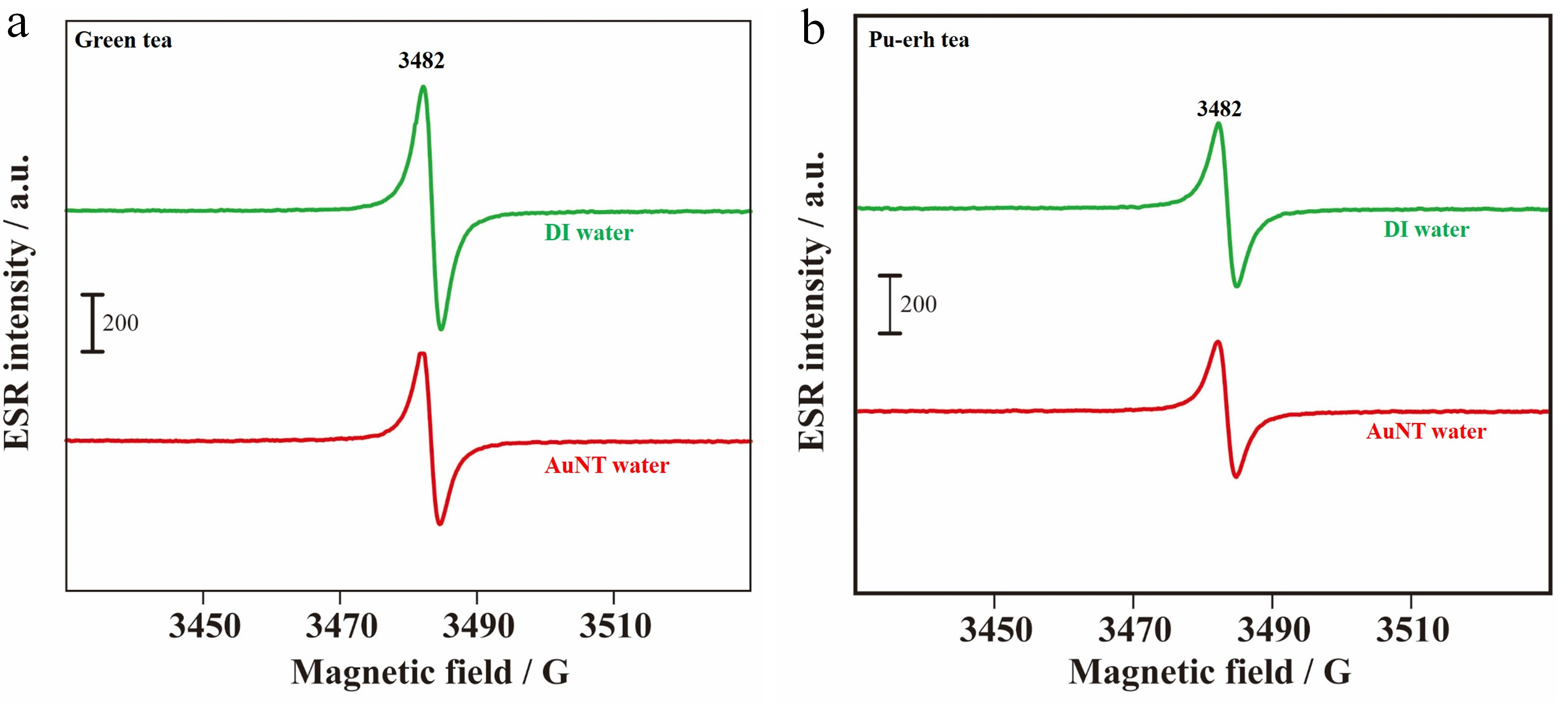


**Figure S3 │** ESR spectra of 2,2-diphenyl-1-picrylhydrazyl (DPPH) free radicals based on solutions of green tea (a) and Pu-er tea (b) extracted with deionized (DI) water and gold nanoparticle-treated (AuNT) water.


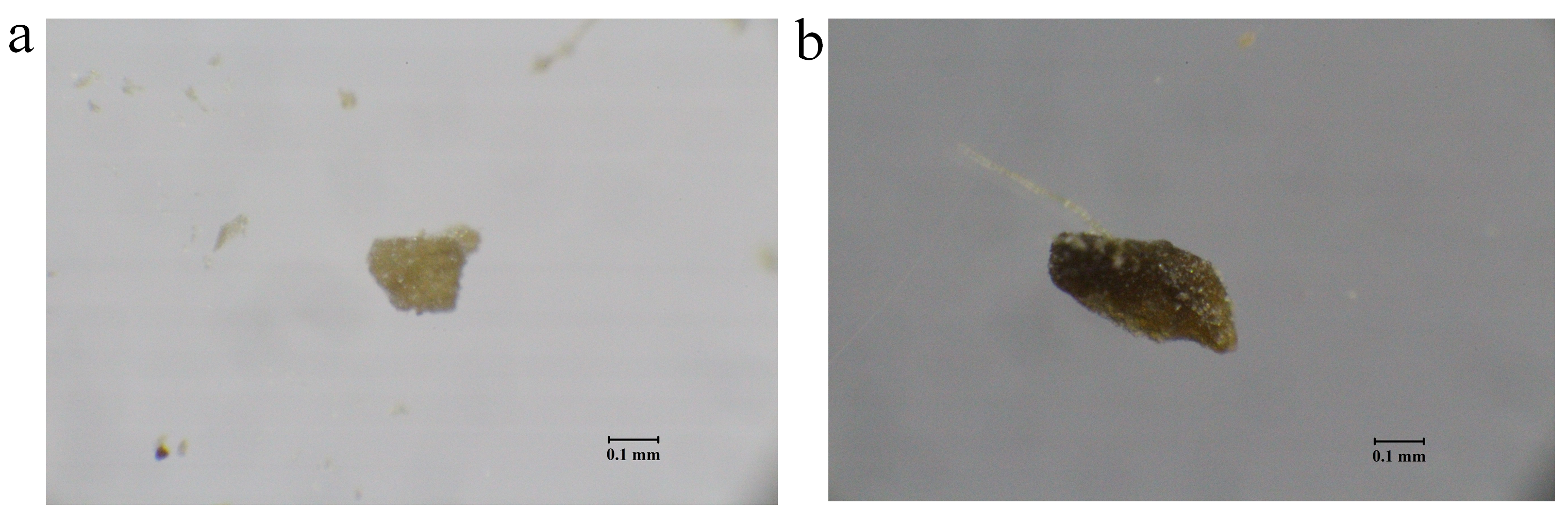


**Figure S4 │** Optical microscopic images of micro-sized powders of green tea (a) and Pu-er tea (b).


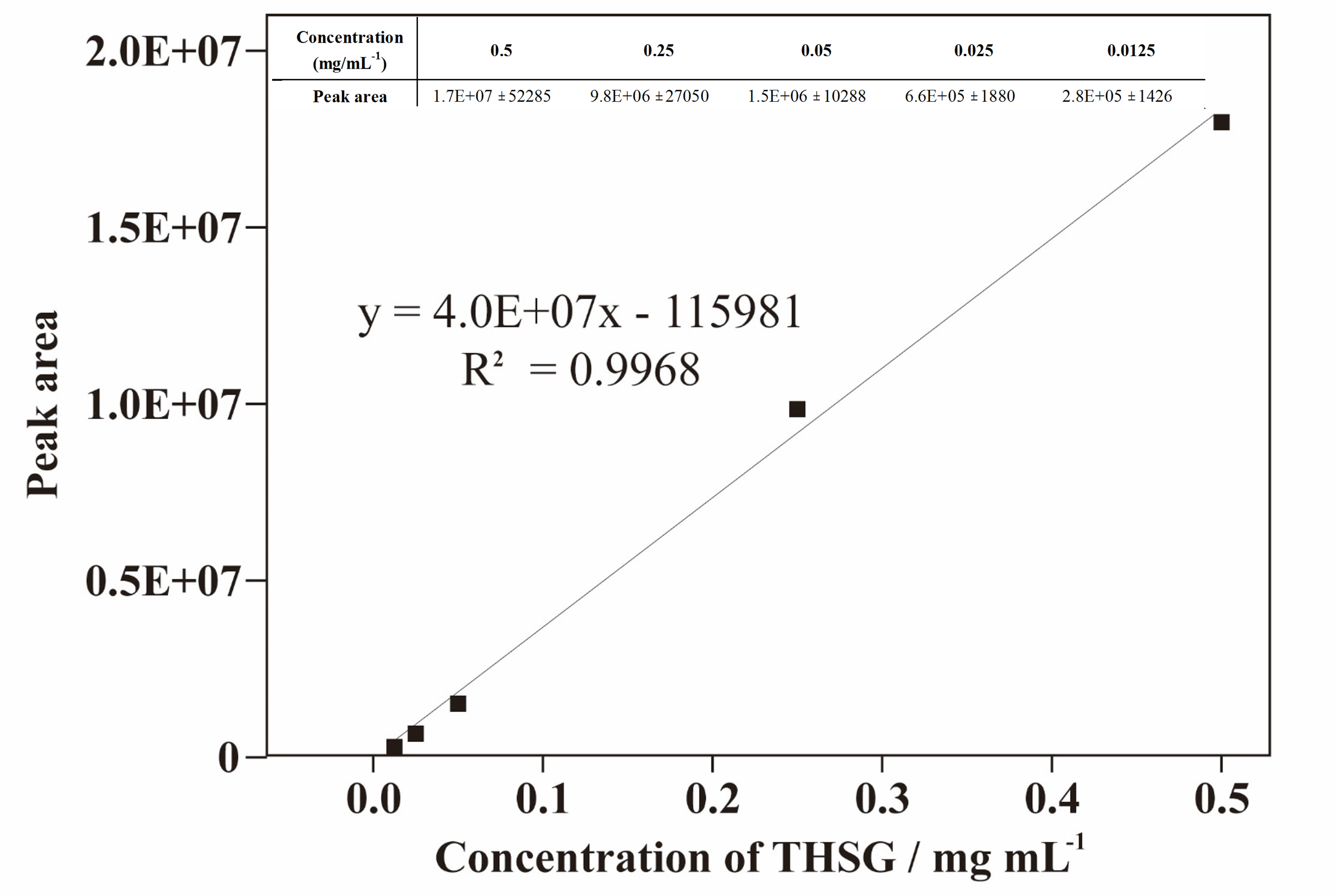


**Figure S5 │** Standard calibration curve of peak area vs. THSG concentration used in the HPLC analyses.


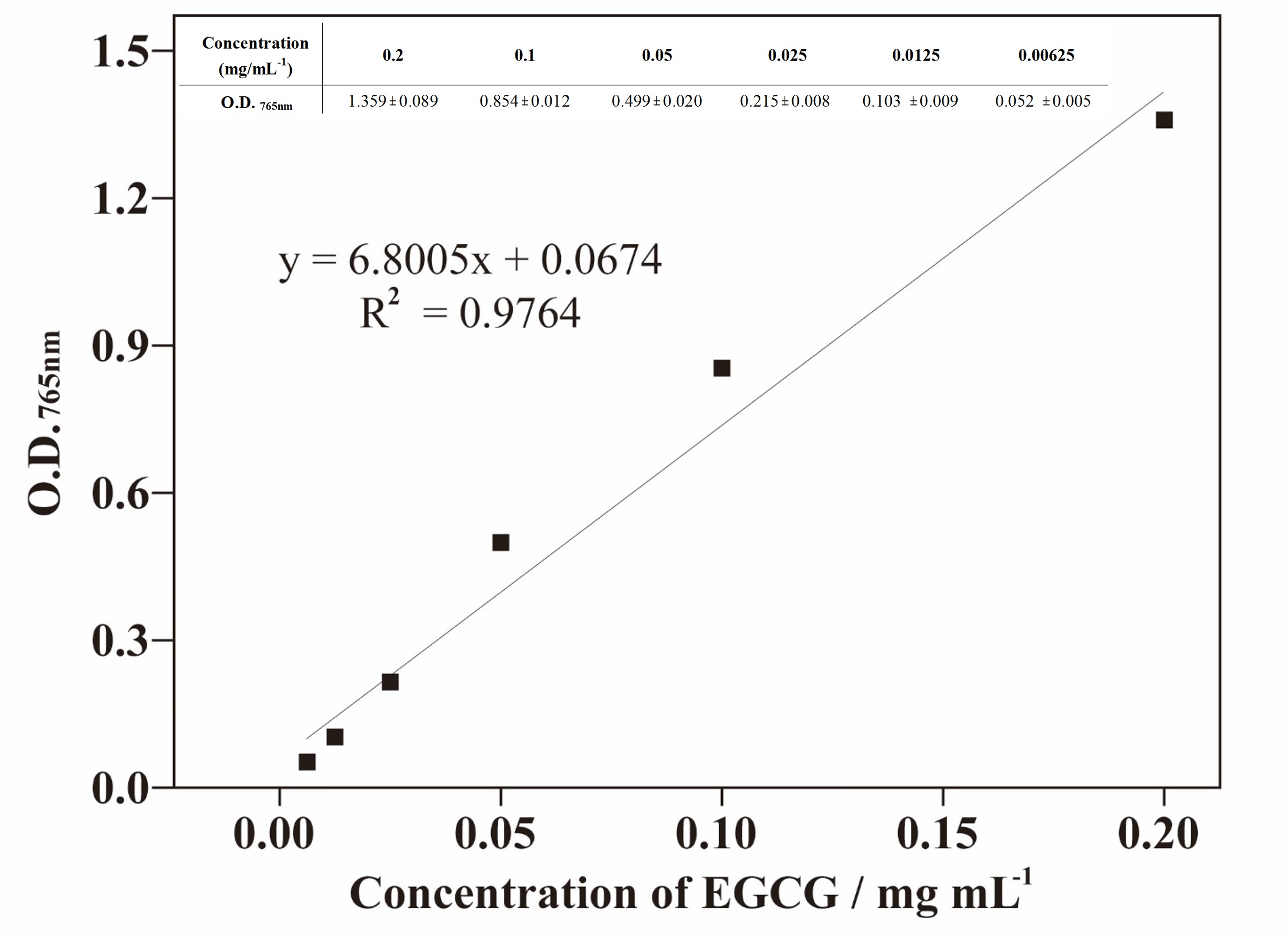


**Figure S6 │** Standard calibration curve of absorbance vs. EGCG concentration used in the UV-vis spectrum analyses.

**References**

1. Zhang, Q. *et al*. A simple 96-well microplate method for estimation of total polyphenol content in seaweeds. *J. Appl. Phycol*. **18**, 445-450 (2006).

2. Huang, Y. S. *et al*. Highly aligned and single-layered hollow fibrous membranes prepared from polyurethane and silica blends through a two-fluid coaxial electrospun process. *Macromol. Chem. Phys*. **215**, 879-887 (2014).
